# Exploring MicroRNA and Exosome Involvement in Malignant Pleural Mesothelioma Drug Response

**DOI:** 10.3390/cancers14194784

**Published:** 2022-09-30

**Authors:** Ben Johnson, Ling Zhuang, Emma M. Rath, Man Lee Yuen, Ngan Ching Cheng, Huaikai Shi, Steven Kao, Glen Reid, Yuen Yee Cheng

**Affiliations:** 1 Asbestos Diseases Research Institute, Sydney, NSW 2139, Australia; 2 Giannoulatou Laboratory, Victor Chang Cardiac Research Institute, Darlinghurst, NSW 2010, Australia; 3 School of Medical Sciences, University of New South Wales, Sydney, NSW 2052, Australia; 4 Chris O’Brien Life House, Sydney, NSW 2050, Australia; 5 Sydney Medical School, The University of Sydney, Sydney, NSW 2006, Australia; 6 Department of Pathology, Otago Medical School, University of Otago, Dunedin 9016, New Zealand

**Keywords:** malignant pleural mesothelioma, microRNA, drug response, exosome, survivin, small molecule inhibitor, chemotherapy drug, inhibitor of apoptosis

## Abstract

**Simple Summary:**

Malignant pleural mesothelioma (MPM) is a deadly thoracic malignancy with limited treatment options. Chemotherapy remains the most widely used first-line treatment for unresectable MPM but is hampered by drug resistance issues. Small molecule inhibitors and microRNA mimics have shown promising potential for the treatment of MPM in preclinical studies, but are yet to be successfully implemented in the clinical setting. Our study aims to provide an understanding of the molecular mechanism(s) that mediate drug response in MPM. The inhibitor of apoptosis family member, survivin, has been reported to be over-expressed in MPM and is associated with drug resistance. Therefore, we particularly focused on determining the cellular mechanism(s) that contribute to MPM cell response to a survivin small molecule inhibitor, YM155. Our study provides key information to facilitate a prediction of the potential utility of small molecule inhibitors and microRNA mimics as treatment options for MPM.

**Abstract:**

Malignant pleural mesothelioma (MPM) is a deadly thoracic malignancy and existing treatment options are limited. Chemotherapy remains the most widely used first-line treatment regimen for patients with unresectable MPM, but is hampered by drug resistance issues. The current study demonstrated a modest enhancement of MPM cell sensitivity to chemotherapy drug treatment following microRNA (miRNA) transfection in MPM cell lines, albeit not for all tested miRNAs. This effect was more pronounced for FAK (PND-1186) small molecule inhibitor treatment; consistent with previously published data. We previously established that MPM response to survivin (YM155) small molecule inhibitor treatment is unrelated to basal survivin expression. Here, we showed that MPM response to YM155 treatment is enhanced following miRNA transfection of YM155-resistant MPM cells. We determined that YM155-resistant MPM cells secrete a higher level of exosomes in comparison to YM155-sensitive MPM cells. Despite this, an exosome inhibitor (GW4896) did not enhance MPM cell sensitivity to YM155. Additionally, our study showed no evidence of a correlation between the mRNA expression of inhibitor of apoptosis (IAP) gene family members and MPM cell sensitivity to YM155. However, two drug transporter genes, *ABCA6* and *ABCA10*, were upregulated in the MPM cell lines and correlated with poor sensitivity to YM155.

## 1. Introduction

Malignant pleural mesothelioma (MPM) is an aggressive tumour of the membrane lining of the lung which is highly related to asbestos exposure. There are limited effective treatment options available to MPM patients in the clinical setting which consequently attributes to a poor associated median survival of 12 to 18 months following first-line chemotherapy treatment with cisplatin and pemetrexed [1,2]. MPM has an extremely poor prognosis and is inherently chemo-resistant. Only approximately 40% of patients respond to the current cisplatin and pemetrexed combination regime [3,4]. The acquisition and maintenance of a chemotherapy-resistant phenotype in MPM represents a major clinical and scientific challenge in the improvement of current therapeutic strategies. Considering this, there is an urgent requirement to uncover the driving molecular pathways and how they are regulated [5]. The exact mechanisms causing drug resistance in MPM are not completely understood and evidence so far suggest that the mechanisms responsible for the resistant phenotype of MPM tumours is likely to be multifaceted.

Tumour resistance to chemotherapy drug treatment can partly be attributed to the tumour microenvironment (TME) protecting the tumour cells against treatment; known as extrinsic resistance. This extrinsic resistance arises from tumour cells interacting with their surrounding environment and can cause alterations in gene expression profiles via the exchange of small RNAs, such as microRNAs (miRNAs) [6,7,8,9]. Exosomes are nanosized membrane vesicles that constitute an extrinsic constituent of the TME that promote therapy resistance via the transport of DNA, RNA (including miRNA), lipids and proteins to recipient tumour cells [10]. Resulting changes in protein expression in the recipient cells can induce an increase in tumour cell survival and DNA repair [8]. Additionally, it has been established that exosomes augment resistance to therapy of the donor cells by reducing intracellular drug concentrations and disposing of pro-apoptotic proteins, such as caspases [11]. Despite exosome involvement in cell transformation and cancer progression being well characterised for other cancer types, such as pancreatic, melanoma, breast and ovarian cancers [12,13,14,15], only limited studies have investigated their involvement in mesothelioma progression [16,17,18]. Furthermore, it has been widely reported that exosome involvement is a contributing factor to tumour resistance to chemotherapy drug treatment [10,19,20], however exosome involvement in MPM chemotherapy resistance is yet to be elucidated.

Survivin is a key member of the inhibitor of apoptosis protein (IAP) family, which mediates the regulation of both apoptosis and cell division [21]. The IAP family of proteins function by binding to and impeding the activity of caspases, leading to a suppression of programmed cell death [22]. Survivin expression is not a typical characteristic of normal differentiated tissues but is frequently overexpressed in several cancers, including MPM [21,23,24,25,26,27]; facilitating cancer progression by enabling tumour cells to bypass apoptotic checkpoints [21]. Given that many anti-tumour agents function via apoptosis activation, it has been proposed that the high tumour-associated survivin expression contributes to drug resistance in cancer [28]. Survivin and other IAP family members have been shown to be over-expressed in mesothelioma and have been suggested to be involved in MPM tumour resistance to chemotherapy drug treatment [24,25,27,29,30,31]. This has prompted researchers to investigate means to inhibit survivin expression in tumour cells, whereby an enhanced tumour response to chemotherapy agents, such as cisplatin, have been demonstrated [32]. It has been established that mesothelioma patients that exhibit progressive disease have high serum levels of survivin, yet the application and efficacy of survivin small molecule inhibitors (e.g., YM155) as a potential treatment for MPM remains to be explored [33].

MiRNAs are small non-coding RNAs that play a role in all essential biological pathways and consequently, their downregulation or dysfunction have been implicated in the development and/or progression of cancer, including MPM [5,34]. It has been established that aberrant miRNA expression is a common occurrence in cancer chemotherapy drug resistance after significant correlations between miRNA expression and potency of anti-cancer agents were identified in a diverse panel of 60 human cell lines (NCI-60) [35]. MiRNA involvement in MPM tumour resistance to chemotherapy treatment has been scarcely explored, however we have previously determined that a downregulation of tumour suppressor miRNA (e.g., miR-15a, miR-16, and miR-34a) in drug-resistant MPM cell lines is associated with anti-apoptotic signalling pathways that facilitate MPM drug resistance, and that a restoration of these miRNAs is capable of sensitising MPM to chemotherapy drug treatment [36]. This concept was also demonstrated in another of our previous studies, whereby artificial restoration of miR-16-5p in vivo and in vitro impeded MPM cell proliferation and tumour growth, and sensitised them to chemotherapy drug treatment with pemetrexed and gemcitabine [37]. This result provided the supportive foundation for our follow-up phase I clinical trial, MesomiR-1; the first and only in-human miRNA study to date, which investigated the safety and optimal dose of a miR-16-based mimic delivered via anti-EGFR antibody-targeted bacterial minicells, dubbed TargomiRs. Results generated from this trial validated the safety of the treatment in all 27 patients, with one patient exhibiting an objective response [38] and stable disease in a further 15 patients [39]. Whilst these results are promising, further investigation into miRNAs in combination with other anti-cancer/sensitising agents is warranted to improve the efficacy of chemotherapy for MPM.

We have previously shown that miRNAs that are associated with the FAK pathway are able to enhance MPM response to FAK small molecule inhibitor treatment with PND-1186, albeit with a broad spectrum of sensitivity across a range of MPM cell lines [40]. Additionally, we demonstrated in vitro differences in response to the small molecule suppressor of survivin, YM155, that were subsequently replicated in an in vivo tumour model. The YM155-resistant MPM cells (MSTO-211H) exhibited reduced intracellular drug concentrations in comparison to YM155-sensitive MPM cells (H226); however, there was no correlation with drug transporters [41]. Furthermore, xenografts derived from the YM155-resistant MPM cells showed no growth inhibition by YM155 during treatment. The purpose of this current study was to assess whether miRNA replacement in MPM cell lines has the capacity to enhance MPM cell response to chemotherapy drug treatment, as well as the survivin small molecule inhibitor (YM155) or FAK inhibitor (PND-1186) targeting agents. We therefore selected miRNAs that have previously been reported to contribute to MPM chemotherapy and small molecule inhibitor drug response by other researchers and from our previously published papers [35,36,42]. Our selection of MPM cell lines for this current study included the previously untested FAK small molecule inhibitor (PND-1186)-resistant MPM cell line, VMC23, and the survivin small molecule inhibitor (YM155)-resistant MPM cell line, MSTO-211H, which were utilized to facilitate an assessment of whether miRNA replacement is capable of enhancing MPM cell response to FAK and survivin small molecule inhibitor treatment, respectively. Additionally, we sought to investigate whether the differing MPM cell response to survivin small molecule inhibitor treatment with YM155 (as previously established for MSTO-211H and H226) is associated with MPM cell-related exosome production and/or aberrant survivin-related gene expression.

## 2. Materials and Methods

### 2.1. Chemicals and Reagents

PND-1186 and YM155 (Sepantronium Bromide) were purchased from Selleck Chemicals. TRIzol was purchased from Life Technologies (Carlsbad, CA, USA). Cisplatin was purchased from McFarlane Medical & Scientific Pty Ltd. (Sydney, Australia) Gemcitabine was purchased from Eli Lilly and Company (Sydney, Australia)

### 2.2. Cell Culture

Five MPM cell lines (H2052, H2452, H28, H226 and MSTO) and the immortalised mesothelial cell line, MeT-5A, were obtained from American Type Culture Collection (ATCC, Manassas, VA, USA). The primary mesothelioma cell line, MM05, was generated at the University of Queensland Thoracic Research Centre (The Prince Charles Hospital, Brisbane), and Ren cells [43] were provided by Laura Moro of the University of Piemonte Orientale A. Avogadro, Novara, Italy. The VMC20, VMC23 and VMC40 MPM cells were kindly provided by Dr Michael Grush from the Medical University of Vienna. All cell lines were maintained in RPMI medium supplemented with 10% fetal calf serum (Life Technologies) at 37 °C, 95% humidity and 5% CO_2_.

### 2.3. MiRNA and SiRNA Transfection

Specific miRNA mimic candidates (Table S1) were selected for testing in combination with either YM155, PND-1186 or chemotherapy drug treatment (i.e., cisplatin and gemcitabine) on the basis of our preliminary target scan analysis of miRNAs corresponding to survivin gene family members (Figure S1), previously studied miRNAs in relation to FAK inhibitor drug response in MPM [40] and miRNAs that have previously been reported to play a role in chemotherapy drug response in MPM [35,36,42]. Additionally, siRNAs specific for the *ABCA6* and *ABCA10* genes were selected to investigate their involvement in MPM cell response to YM155 treatment (Table S2). All miRNA mimics and siRNAs were purchased from Shanghai GenePharma, and transfection reagents were purchased from Life Technologies. A reverse transfection of the different miRNA mimics (1 nM) (including an inactive in-house miRNA control mimic [44]) or siRNAs (5 nM), was performed following seeding of the MPM cells at a density of 2500 cells in 100 μL medium per well. Lipofectamine RNAiMax (LRM), at a concentration suggested by the manufacturer’s user manual, was utilized to introduce the miRNAs or siRNAs into the MPM cell lines.

### 2.4. Reverse Transcription and Quantitative Real Time PCR (RT-qPCR) Quantification

Total RNA was extracted from the MPM and MeT-5A cell lines using Trizol reagent (Life Technologies) according to the manufacturer’s instructions. Reverse transcription (RT) reactions were performed using 200 ng of total RNA with an MMLV first strand cDNA kit (Promega, Madison, WI, USA) according to the manufacturer’s protocol. The expression of the IAP and drug transporter (*ABCB1*, *ABCG2*, *ABCA6*, *ABCA10, BIRC5, OCT1, MRP1, IAP2, ILF3* and *XIAP)* mRNA was determined by quantitative real time PCR (RT-qPCR) using KAPA SYBR^®^ FAST reagents (Sigma, Cape Town, SA, USA) and the Viia7 qPCR System (ABI QuantStudio^TM^ Real-Time PCR Software v.1.1). Probe Design software (Roche Diagnostics Australia) was used for designing PCR primers (Table S3). 18S was used as a reference gene. mRNA expression was presented as fold change, relative to the mRNA expression of the MeT-5A cells, for each tested cell line. Relative quantification was performed as described previously [45].

Following a 24 h incubation period after miRNA mimic transfection of the MSTO-211H and VMC23 cells, total RNA was extracted using Trizol reagent (Life Technologies) according to the manufacturer’s protocol. For miRNA quantification experiments, RT reactions were performed using 50 ng of total RNA with a TaqMan microRNA reverse transcription kit (Thermo Fisher Scientific, Vilnius, LT, USA) in accordance with the manufacturer’s protocol. The miRNA expression was determined by RT-qPCR using KAPA PROBE FAST (Sigma, Cape Town, SA, USA) and miRNA-specific TaqMan assay (Thermo Fisher Scientific, Pleasanton, CA, USA) reagents (Table S1) using the Viia7 qPCR system (ABI QuantStudio^TM^ Real-Time PCR Software v.1.1). *RNU6B* was used as an endogenous control. Post-transfection miRNA expression was calculated as fold change, relative to the miRNA expression of the untransfected control MSTO-211H and VMC23 cells, for each tested miRNA mimic. *BIRC5* (survivin) and *PTK2* (FAK) RT and RT-qPCR quantification was carried out on the miRNA-transfected MSTO-211H and VMC23 cells using the MMLV cDNA kit and KAPA SYBR^®^ FAST methods, respectively, as described above. *BIRC5* and *PTK2* mRNA expression was determined as fold change, relative to the mRNA expression of the untransfected control cells, for each tested miRNA mimic.

### 2.5. Immunohistochemical Analyses of Survivin and FAK Expression in MiRNA-Transfected MPM Cells

Following a 24 h incubation period after miRNA mimic transfection of the MSTO-211H and VMC23 cells, the cells were fixed in 10% formalin and processed into paraffin-embedded cell blocks. The cell blocks were prepared as 5 µm sections and deparaffinized in three changes of xylene (3 min each), followed by three changes of 100% ethanol (3 min each) and three changes of 70% ethanol (3 min each). The sections were incubated with a citrate buffer (10 mM, PH = 6) for 1 h at 86 °C and then stained with either anti-FAK (1:100, Abcam) or anti-survivin (1:500, Abcam) antibodies overnight at 4 °C. The sections were then subjected to 150 µL of secondary antibody (VECTASTAIN^®^ Elite ABC Kit, rabbit, 1:200) for 60 min at room temperature, followed by Avidin-Biotin Complex (ABC, VECTASTAIN^®^ Elite ABC Kit) for 30 min. Visualization of cells with bound primary antibody was performed by exposing them to diaminobenzidine chromogenic (DAB) substrate for 1 min, followed by counterstaining with 10% hematoxylin for 3 min. All slides were mounted with coverslip and air dried overnight before imaging with a ZEISS Axio.M2 microscope with the 20× objective lens.

### 2.6. Drug Treatment and Proliferation Assay

MPM cell response to drug treatment was assessed by the Alamar Blue cell death assay in 96-well plates with various concentrations of chemotherapeutic drugs and small molecule inhibitors. Briefly, following a 24 h incubation period after miRNA or siRNA transfection, cells were treated with a medium containing various drug concentrations of either cisplatin, gemcitabine, PND-1186 or YM155. In addition to the chemotherapy drugs and small molecule inhibitors, the exosome inhibitor, GW4869, was added to study the MPM drug response in relation to exosome inhibition. Following drug treatment, the cells were incubated for 72 h. Alamar Blue was prepared as previously described [46]; filter-sterilised and stored at 4 °C in the dark. Following the 72 h incubation, Alamar Blue was added (1:10) to the cells and incubated for 4 h at 37 °C. Fluorescence intensity was measured at 590–10 nm with 544 nm excitation, using a FLUOstar Optima plate reader (BMG LabTech, Ortenberg, Germany). Fluorescence intensity was presented as a percentage of intensity with respect to the untreated control cells. The untreated control cells were normalized to a cell viability of 100% to account for any cell viability loss induced by the miRNA or siRNA pre-treatment prior to carrying out drug IC_50_ calculations (the inhibitory concentration at which 50% cell viability is lost). Experiments involving human cell lines were performed 3 times with 3 replicates each time.

### 2.7. Exosome Isolation

2 × 10^6^ MPM cells were seeded in a T75 flask with 10 mL growth media (containing 10% exosome depleted FCS). At 48 h post cell seeding, 10 mL of conditioned media was collected from each MPM cell line. Exosomes were isolated according to the manufacturer’s instruction using a total exosome isolation kit (Invitrogen, Cat. Number 4478359). Exosome protein analysis was performed using the Bradford assay.

### 2.8. Statistical Analysis and Drug IC_50_ Modelling

To assess the statistical significance between the miRNA mimic-treated MPM cell proliferation and the inactive miRNA control mimic-treated MPM cell proliferation, the following method was applied as previously described [46]. MPM cell responses to treatment were modelled using a sigmoid function [47]. Briefly, the sigmoid function used to predict MPM cell proliferation, *y*, was:

$$y=A+\left(B-A\right)*\frac{1}{\left(1+\exp\left(\frac{\left(xmid+xshift-x\right)}{scale}\right)\right)}$$

In the above formula, A is the left asymptote (MPM cell response at drug treatment concentration of 0), B is the right asymptote (MPM cell response at highest drug treatment concentration), xmid is the transition point (IC_50_) of the miRNA control mimic-treated MPM cells (one component model), xmid+xshift is the transition point (IC_50_) for the miRNA mimic-treated MPM cells (two component model), scale is an x-axis scale parameter impacting slope of the transition, and x is log10 of the drug treatment concentration (thus rendering the curve symmetrical and suitable for modelling using log-likelihood). The best fitting parameters for a given model were determined by the maximum log likelihood method, using the optimx package [48] in R [49]. The likelihood ratio test was used to compare two competing models where the models are nested (i.e., the one component model is obtained from the two component model by removing the xshift parameter). The chi-squared statistic was used to determine whether the improvement of fit for the two component model over the one component model is statistically significant (*p*-value < 0.05), using the following formulae for chi-squared value and degrees of freedom:

$${{Χ}^{2}}_{difference\_in\_models}={{Χ}^{2}}_{2\_component\_model}-{{Χ}^{2}}_{1\_component\_model}$$

$$df_{\;difference\_in\_models}=df_{2\_component\_model}-df_{1\_component\_model}$$

IC_50_ was calculated as the sigmoidal transition point resulting from the two component model having the best fit (lowest *p*-value). Fold change was calculated as the control’s IC_50_ divided by the drug’s IC_50_. The one component model (without the xshift parameter) was used to calculate the IC_50′_s for the siRNA-treated VMC23 MPM cells. IC_50_ standard deviation was calculated as the standard deviation of the transition points for each experiment modelled individually as a sigmoid function, as shown in detail in the R code text file (Figure S2). To determine whether up- or downregulated mRNA expression levels of genes or exosome production levels in the MPM cell lines were statistically significant in comparison to the non-malignant MeT-5A mesothelial control cell line, the Welch Two Sample *t*-test in R was used. Pearson’s product moment correlation coefficient implemented in the R cor.test function was used to determine whether there was a correlation between gene expression (mRNA) and the IC_50_ for each MPM cell line, as shown in detail in the R code text file (Figure S2).

## 3. Results

### 3.1. MicroRNA Restoration Sensitizes MPM Cells to FAK Inhibitor (PND-1186) and Survivin Inhibitor (YM155) Treatment

**Figure 1:**
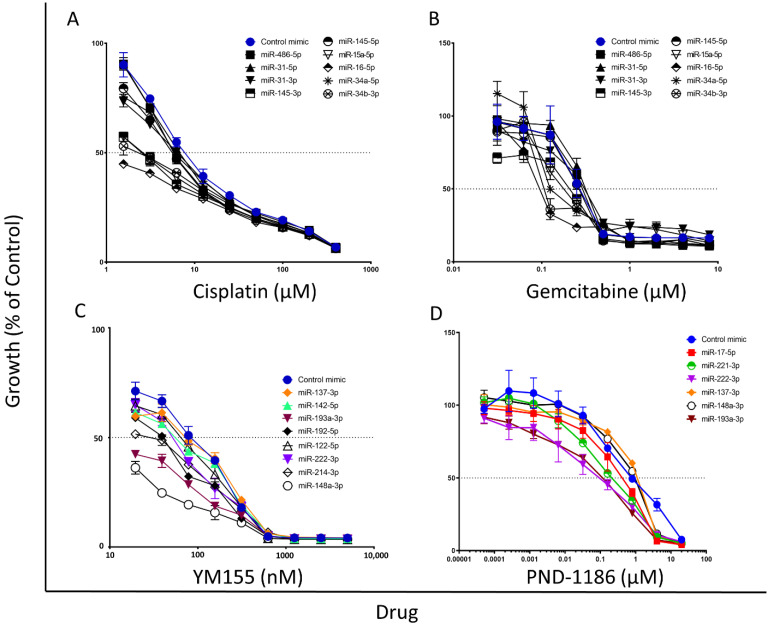
Dose response curves depicting an enhanced MPM cell sensitivity to drug treatment with (**A**) cisplatin, (**B**) gemcitabine, (**C**) YM155 and (**D**) PND-1186, following transfection with various miRNAs (1 nM). The FAK inhibitor-resistant VMC23 cell line was used for all experiments involving PND-1186 treatment. The chemotherapy drug and survivin small molecule inhibitor-resistant MSTO-211H cell line was used for all experiments involving cisplatin, gemcitabine and YM155 treatment. All dose responses are shown with respect to the miRNA control mimic. Error bars represent the mean ± SD, as determined from three experimental replicates per tested drug concentration.

**Figure 2:**
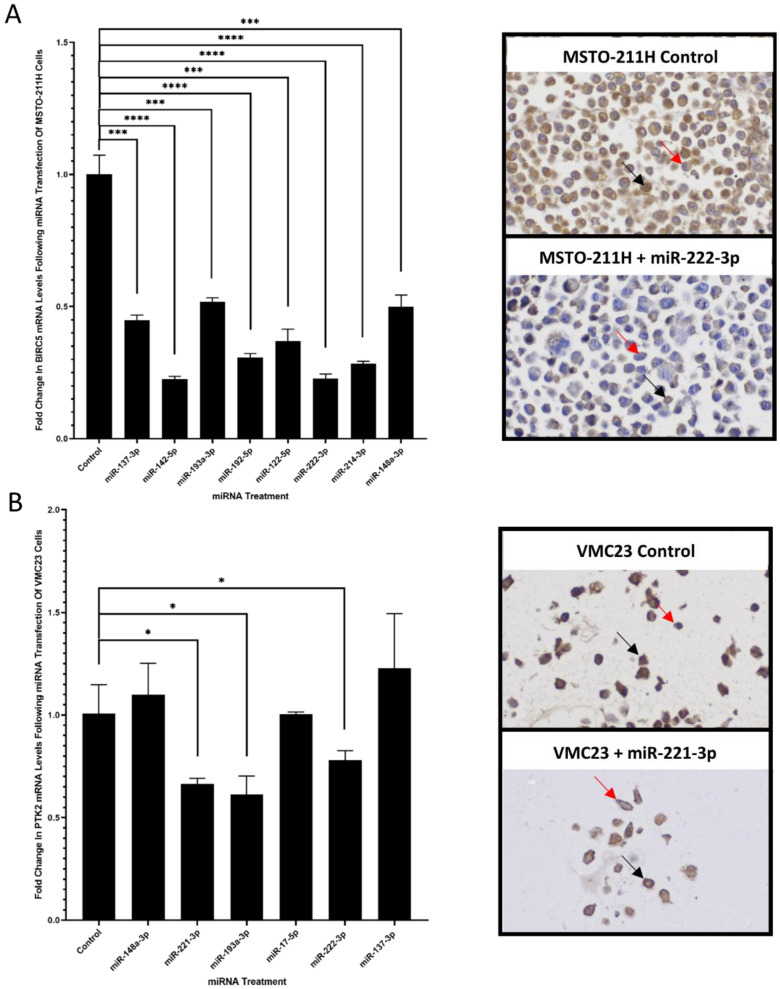
Graphs depicting the fold change in expression of (**A**) BIRC5 (survivin) and (**B**) PTK2 (FAK) mRNA levels following 24 h of 1 nM miRNA transfection of MSTO-211H and VMC23 cells, respectively. The *BIRC5* and *PTK2* mRNA levels were quantified via RT-PCR with respect to the untransfected control cells (normalised to a value of 1). Error bars represent the mean ± SD, as determined from three experimental replicates. Statistically significant fold changes in expression were determined via a Student’s *t*-test, whereby a *p*-value of ≤0.05, ≤0.0005 and ≤0.00005 is indicated on the graphs as *, ***, and ****, respectively. Representative images of IHC stained cells are shown for MSTO-211H post-miR-222-3p transfection and VMC23 post-miR-221-3p transfection in comparison to their respective untransfected controls. Survivin/FAK-positive (brown) and -negative (purple) cells are indicated with black and red arrows, respectively. Images were captured with a ZEISS Axio.M2 microscope with 20× objective.

In this study we determined whether miRNAs have the capacity to sensitise MPM cells to treatment with cisplatin and gemcitabine chemotherapy drugs, FAK small molecule inhibitor treatment with PND-1186, and survivin small molecule inhibitor treatment with YM155. All tested miRNA mimics induced a statistically significant increase in miRNA expression in the MSTO-211H cells (with respect to the untransfected control cells) following miRNA transfection (Figure S3A). Our results demonstrated that four of the tested miRNAs (miR-145-3p, miR-15a-5p, miR-16-5p, and miR34b-3p) increased MSTO-211H sensitivity to chemotherapy drug treatment with cisplatin by 3-fold or more compared to the inactive miRNA control mimic (Figure 1A; Table 1 and Table S4). The other tested miRNAs (miR-486-5p, miR-31-3p, miR-31-5p, miR-145-5p, and miR-34a-5p) exhibited a modest increase in MSTO-211H sensitivity to cisplatin treatment by less than 2-fold (Figure 1A; Table 1 and Table S4). Three of the tested miRNAs (miR-16-5p, miR34a-5p, and miR-34b-3p) were found to induce an increased MSTO-211H sensitivity to chemotherapy drug treatment with gemcitabine by 2-fold or more with respect to the inactive miRNA control mimic (Figure 1B; Table 1 and Table S4). Two of the miRNAs (miR-145-3p and miR-15a-5p) induced a modest increase in MSTO-211H cell sensitivity to gemcitabine treatment and the remaining four miRNAs (miR-486-5p, miR-31-3p, miR-145-5p, and miR-31-5p) showed no increase in MSTO-211H sensitivity to gemcitabine treatment (Figure 1B; Table 1 and Table S4). We tested a range of miRNAs that contribute to the survivin pathway to explore their involvement in MPM cell response to the survivin small molecule inhibitor, YM155 (Figure S1). All of the tested miRNA mimics induced an evident decrease in survivin mRNA (*BIRC5*) and survivin protein expression in the MSTO-211H cells, with respect to the untransfected control cells, following miRNA transfection (Figure 2A) (Five of the tested miRNAs (miR-222-3p, miR-148a-3p, miR-193a-3p, miR-192-5p, and miR-214-3p) induced an increased MSTO-211H cell sensitivity to YM155 treatment with respect to the inactive miRNA control mimic (Figure 1C; Table 1 and Table S4). In particular, miR-148a-3p and miR-193a-3p increased MSTO-211H sensitivity to YM155 treatment by more than 5-fold, whereas miR-222-3p, miR-192-5p and miR-214-3p induced a modest increase in MSTO-211H sensitivity to YM155 treatment by less than 2-fold (Table 1). The three other tested miRNAs (miR-137-3p, miR-142-5p and miR-122-5p) did not enhance MSTO-211H sensitivity to YM155 treatment (Figure 1C; Table 1 and Table S4). Additionally, we investigated miRNA candidates in relation to MPM cell response to FAK inhibitor (PND-1186) treatment using the previously untested FAK small molecule inhibitor-resistant VMC23 MPM cell line. All tested miRNA mimics induced a statistically significant increase in miRNA expression in the VMC23 cells, with respect to the untransfected control cells, following miRNA transfection (Figure S3B). The VMC23 cells exhibited an increased sensitivity to PND-1186 for four of the tested miRNAs (miR-17-5p, miR-221-3p, miR-222-3p, and miR-193a-3p) in comparison to the inactive miRNA control mimic (Figure 1D, Table 1 and Table S4), which was associated with a modest decrease in FAK mRNA (*PTK2*) expression (with the exception of miR-17-5p), with respect to the untransfected control cells; albeit a decrease in FAK protein expression was less apparent (Figure 2B). Pre-treatment with miR-222-3p and miR-193a-3p in particular, induced a 12-fold and 10-fold increase in VMC23 cell sensitivity to PND-1186 treatment in comparison to the inactive miRNA control mimic, respectively (Table 1). The other two tested miRNAs (miR-137-3p and miR-148a-3p) did not enhance VMC23 cell sensitivity to PND-1186 treatment (Figure 1D, Table 1 and Table S4) and did not induce an evident reduction in the FAK mRNA (*PTK2*) and FAK protein expression, with respect to the untransfected control cells, following miRNA transfection (Figure 2B).

### 3.2. MicroRNA Variably Sensitizes MPM Cells to Survivin Inhibitor (YM155) Treatment and Is Unrelated to Exosome Involvement

**Figure 3:**
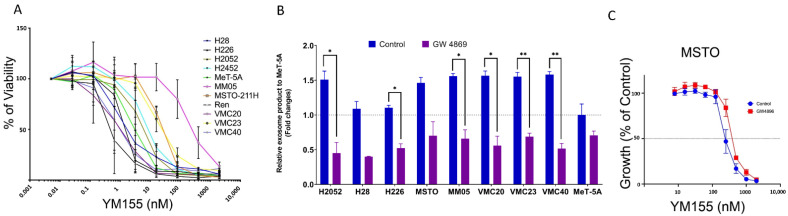
Graphs depicting (**A**) MPM cell response to increasing concentrations of YM155 treatment; (**B**) quantified levels of exosome production in conditioned medium obtained from MPM cell cultures, with and without exosome inhibitor (GW4869), determined using an exosome prep kit; and (**C**) MSTO-211H response to increasing concentrations of YM155 following pre-treatment of MSTO-211H cells with the GW4896 exosome inhibitor, with respect to MSTO-211H cells not pre-treated with GW4896. Error bars represent the mean ± SD, as determined from three experimental replicates. Statistically significant differences in exosome production between MPM cells treated with and without exosome inhibitor (GW4869) are indicated with a single asterisk (*) for a *p*-value of ≤0.05 and with a double asterisk (**) for a *p*-value of ≤0.01.

Mechanisms of MPM response to chemotherapy drug and FAK (PND-1186) small molecule inhibitor treatment were studied in our previous papers [36,40]. In this study, we particularly focused on studying the mechanisms that contribute to MPM response to survivin small molecule inhibitor treatment (YM155). We first surveyed MPM cell response to YM155 treatment using a variety of MPM cell lines and the non-malignant MeT-5A mesothelial cell line control. This revealed that the MPM cell lines exhibit a broad spectrum of responses following treatment with the YM155 small molecule inhibitor; with some MPM cell lines exhibiting a greater sensitivity to YM155 treatment than others (Figure 3A), and with IC_50′_s ranging from 1 nM (Ren) to 300 nM (MM05).

We assessed whether exosome production potentially contributes to this variable MPM cell response to survivin small molecule inhibitor treatment by collecting conditioned media from a range of cultured MPM cell lines and performing a subsequent exosome prep and quantification analysis. Our results show that all tested MPM cell lines secreted a higher level of exosomes in comparison to the non-malignant Met-5A mesothelial cell control and that there was a statistically significant reduction in exosome secretion following treatment with the GW4896 exosome inhibitor for all tested MPM cell lines, with the exception of H28 and MSTO-211H (Figure 3B). Additionally, it was determined that some of the tested MPM cell lines (MSTO-211H, H2052, MM05, VMC20, VMC23 and VMC40) secreted relatively higher levels of exosomes than others (H28 and H226) (Figure 3B). The YM155-resistant MSTO-211H cell line was selected for a subsequent experiment to determine whether the GW4896-induced suppression of exosome production could potentially enhance sensitivity of the MSTO-211H cells to YM155 small molecule inhibitor treatment. Pre-treating the MSTO-211H cells with the exosome inhibitor, GW4896, did not result in a statistically significant difference in sensitivity to YM155 treatment in comparison to the untreated control MSTO-211H cells (Figure 3C; *p*-value = 1).

### 3.3. Drug Transport-Related Genetic Alterations Contribute to MPM Cell Response to Survivin Inhibitor (YM155) Treatment

**Figure 4:**
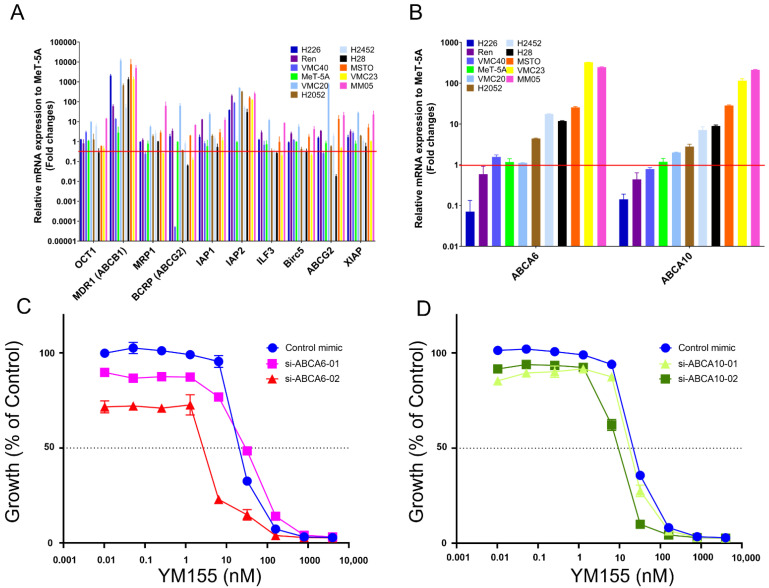
Graphs depicting (**A**) mRNA expression levels corresponding to genes known to play a role in chemotherapy drug resistance, relative to the non-malignant MeT-5A control, as determined via qPCR analysis on a range of MPM cell lines; (**B**) mRNA levels corresponding to the *ABCA6* and *ABCA10* genes, relative to the non-malignant MeT-5A control, as determined via qPCR analysis on a range of MPM cell lines; MSTO-211H growth response to increasing concentrations of YM155 following silencing of the (**C**) *ABCA6* and (**D**) *ABCA10* genes. The dose response curves of the siRNA-treated cells are shown with respect to the untreated miRNA control mimic. Error bars represent the mean ± SD, as determined from three experimental replicates.

Upon establishing that there was no evident link between exosome secretion and MPM sensitivity to YM155 small molecule inhibitor treatment, we alternatively suspected that survivin-related gene expression may play a role. To determine whether survivin-related genetic alterations play a role in small molecule inhibitor drug response in MPM, we examined the gene expression levels of the IAP family members, including transporters involved in the cellular uptake and efflux of drug molecules, and correlated their expression with MPM cell response to YM155 small molecule inhibitor treatment. In particular, we assessed the expression of *BIRC5, XIAP, IAP1* and *IAP2* members of the IAP gene family; the drug-transporting ABC transporters, MDR1 (*ABCB1*), MRP1 (*ABCC1*) and BCRP (*ABCG2*); the uptake transporters, OCT1 (*SLC22A1*); and the survivin regulator, ILF3 (*ILF3*). Upon conducting qPCR analyses to examine the mRNA levels of these genes in MPM cell lines, our data indicated that MDR1 (*ABCB1*) and *IAP2* (genes known to mediate drug transport and anti-apoptosis [21,50], respectively) are consistently upregulated in the tested MPM cell lines in comparison to the non-malignant mesothelial MeT-5A control (Figure 4A). The *IAP2* and MDR1 (ABCB1) mRNA levels were found to be approximately 30- to 503-fold and 14- to 11,897-fold higher than the MeT-5A control, respectively, (Table S5); albeit only *IAP2* upregulation was determined to be statistically significant (Table S6; Welch Two Sample *t*-test *p*-value = 0.02598). In contrast, OCT1 (*SLC22A1*), BCRP (*ABCG2*), and *BIRC5* mRNA levels were found to be less than 1-fold for the majority of tested MPM cell lines with respect to the MeT-5A control (Table S5), attributing to a statistically significant downregulation (Table S6; Welch Two Sample *t*-test *p*-value = 0.00819, 0.00829, and 4.386 × 10^−6^, respectively). There was no evident correlation between the up- or downregulated expression of these genes and MPM cell response to YM155 treatment (Pearson’s correlation coefficient *p*-value > 0.05 for each gene).

We examined the gene expression of two drug transporter genes, *ABCA6* and *ABCA10,* in the MPM cells. The qPCR data revealed that *ABCA6* and *ABCA10* are upregulated in the majority of tested MPM cell lines with respect to the non-malignant mesothelial MeT-5A cell control (Figure 4B). *ABCA6* and *ABCA10* mRNA levels were found to range from below 2-fold to more than 200-fold higher in comparison to the MeT-5A control for all tested MPM cell lines, with the exception of H226, Ren and VMC40 (Table S5). There was a strong correlation between the fold-change in expression of *ABCA6* and MPM cell response (IC_50_) to small molecule inhibitor treatment with YM155 (Figure 3A; Pearson’s correlation coefficient = 0.61, *p*-value = 0.00247). There was an even stronger correlation between the fold-change in expression of *ABCA10* and MPM cell response (IC_50_) to small molecule inhibitor treatment with YM155 (Figure 3A; Pearson’s correlation coefficient = 0.91, *p*-value = 5.804 × 10^−9^). The greater the increased expression of *ABCA6* or *ABCA10,* the greater the MPM cell line’s resistance to YM155 treatment. Furthermore, we showed that siRNA treatment of the drug-resistant MSTO-211H cell line induced a reduction in the expression of the *ABCA6* and *ABCA10* genes (Figure S4), which was associated with a notable sensitisation of the MSTO-211H cells to YM155 treatment (Figure 4C,D). The siRNA treatments with either si-ABCA6-02 or si-ABCA10-02 induced a statistically significant sensitisation of the MSTO-211H cells to YM155 treatment in comparison to the inactive miRNA control mimic-treated cells, whereas treatment with si-ABCA6-01 or si-ABCA10-01 did not (Table S7).

The microRNAs that could potentially interact with 3′UTR of mRNA corresponding to genes *XIAP*, *ABCA6*, *ABCA10* and *BIRC5* (Figure S1) were studied. Our results indicated that almost all of the selected aforementioned gene-related miRNAs are able to induce MPM cell sensitivity to YM155 in a resistant cell line (MSTO-211H) (Figure 1C). In particular, the restoration of miR-148a-3p and miR-193a-3p induced the greatest sensitivity to YM155 treatment, correlating to reduced YM155 IC_50_ values of approximately 19 nM and 21 nM (with respect to the inactive miRNA control mimic IC_50_ of approximately 117 nM), respectively (Table 1). Collectively, these results support the potential utility of miRNA to sensitise MPM cells to survivin small molecule inhibitor treatment.

## 4. Discussion

The treatment of mesothelioma in the clinical setting is associated with poor prognosis. Only about 40% of patients diagnosed with this aggressive thoracic cancer respond to the gold standard first-line chemotherapy treatment regimen involving a combination of cisplatin and pemetrexed [3,4]. This is attributed to MPM being notoriously chemo-resistant and consequently results in a poor median patient survival of 12–18 following diagnosis [1,2]. It has been suggested that MPM tumour resistance to chemotherapy drugs is complex and multi-faceted and studies are yet to completely determine the precise intrinsic cellular mechanisms that mediate MPM drug resistance. Survivin over-expression, loss of tumour-suppressor miRNA and exosome involvement are known to contribute to drug resistance for a number of cancer types [20,51,52]. However, whether these factors contribute to chemotherapy and/or small molecule inhibitor drug-resistance in MPM remains to be elucidated. Therefore, in this study we investigated the role of survivin, exosomes and tumour-suppressor miRNAs in relation to MPM cell response to chemotherapy and small molecule inhibitor targeting agents.

Loss of tumour-suppressor miRNA expression is a frequently reported occurrence in MPM [34,53,54], however their contribution to chemotherapy drug response in MPM has been scarcely explored [36,37]. Our results demonstrated a modest enhancement of MPM cell sensitivity to chemotherapy drug treatment with cisplatin and gemcitabine upon restoring tumour-suppressor miRNA expression in the drug-resistant MPM cell line, MSTO-211H (Figure 1A,B). These results are concordant with other studies indicating an enhanced tumour cell sensitivity following tumour-suppressor miRNA restoration [55,56] and collectively suggest that a loss of tumour-suppressor miRNA expression contributes to MPM response to chemotherapy drug treatment. In our study, some (but not all) of the tested miRNAs were found to induce an enhanced sensitivity to chemotherapy drug treatment. We found that MPM cell sensitivity to cisplatin or gemcitabine was enhanced by pre-treatment with miR-145-3p, miR-15a-5p, miR16-5p, miR-34b-3p or miR-34a-5p (Figure 1A,B; Table 1). For other miRNAs, we observed a modest reduction in MPM cell sensitivity, which indicates that in the case of MPM, the expression of certain miRNAs may in fact promote MPM resistance to chemotherapy. For example, our data demonstrated that miR-31-3p and miR-31-5p restoration induced a modest reduction in MPM cell sensitivity to gemcitabine (Figure 1B; Table 1). This result is concordant with the findings of a study by Moody et al., which reported that restoration of miR-31 in an MPM cell line correlates with increased chemoresistance; thus suggesting that certain miRNA loss in MPM may actually promote a beneficial chemosensitive response [5].

In many cancers (including MPM), FAK over-expression has been linked to aggressive tumour behaviour, tumour survival and progression [57,58,59,60]. In this current study, an enhanced MPM cell sensitivity to the FAK inhibitor drug, PND-1186, was observed following miRNA restoration for all tested miRNAs and sensitivity was an order of magnitude higher for two of the tested miRNAs (miR-222-3p and miR-193a-3p) (Figure 1D; Table 1). This data suggests that FAK over-expression and a dysregulation of miRNA expression in MPM may be linked and thus, collectively contribute to MPM chemotherapy drug response. This finding is consistent with recently published data whereby the restoration of several miRNAs known to be downregulated in MPM, induced an enhanced MPM cell sensitivity to FAK inhibitor treatment with PND-1186 [40]. Despite this, the use of FAK inhibitors in clinical trials has demonstrated modest efficacy in MPM patients in terms of promoting an improved progression-free survival (PFS) and disease stabilisation; only exhibiting an objective response in patients with Merlin-negative tumours [61,62]. Our findings from this current study suggests that a treatment regimen involving replacement of the downregulated miRNAs with functional miRNA mimics may potentially be the key to further sensitise MPM tumours to FAK inhibitor treatment and warrants further investigation in prospective preclinical and clinical studies of MPM.

Survivin is an attractive potential target for cancer therapies because it is rarely expressed in normal cells but remarkably upregulated in several types of cancers. YM155 is a selective small molecule inhibitor of survivin transcription that exhibits potent antitumor activity by inducing apoptosis and autophagy in various types of cancer, as well as enhancing the anti-tumour and anti-angiogenic effects of chemotherapy drugs such as cisplatin [63,64,65]. In this present study we demonstrated that YM155 exhibits a broad spectrum of cytotoxic activity across all tested 11 MPM cell lines (Figure 3A) and that MPM cell sensitivity to YM155 treatment is enhanced upon tumour-suppressor miRNA restoration (particularly for miR-148a-3p and miR-193a-3p) (Figure 1C; Table 1). Survivin expression is regulated by miRNAs that target the 3′-UTR of survivin mRNA [66]. In MPM, many of these survivin-regulating miRNAs are downregulated; such as miR-16, miR-203, miR-218 and miR-34a [67]. This reinforces that both a loss of tumour-suppressor miRNAs and survivin over-expression collectively contribute to drug resistance in MPM. Therefore, the potential development of combinational therapies involving the use of small molecule inhibitors of survivin, in combination with miRNA replacement is a strategy that warrants investigation as a potential treatment option for MPM. Additional in vitro and in vivo studies will be needed to assess whether a miRNA-small molecule inhibitor and chemotherapy drug combination approach will improve treatment efficacy for MPM. This treatment strategy has already been explored in preclinical investigations for other cancer types, such as ovarian cancer, and has shown promising efficacy [68]. Consistent with these previous in vivo studies, we previously showed that YM155 significantly impeded in vivo tumour growth during the treatment period of the H226-derived tumour-bearing mice. However, this anti-tumour effect was not sustained once treatment was ceased [41]. Collectively, these results highlight YM155′s potential for treatment of MPM. However, it will be necessary to explore other treatment options in combination with YM155 to potentially induce a more enduring anti-tumour effect that extends beyond the active treatment period. Other studies have demonstrated good efficacy of YM155 treatment in combination with chemotherapy for other cancer types, such as retinoblastoma [69], as well as a favourable safety profile in non-small cell lung cancer patients treated with YM155 in combination with carboplatin and paclitaxel [70]. Findings from our current study indicate that a prospective in vivo study exploring a YM155 an-edited

d miRNA combinational treatment approach on MPM tumour-bearing mice is greatly warranted.

Exosomes have been widely reported to play a key role in chemotherapy drug resistance for a range of cancer types [20] and their possible involvement in MPM chemotherapy resistance has so far been overlooked. Our results indicated that all tested MPM cell lines showed a higher level of exosome production in comparison to the normal mesothelial, MeT-5A cell control (Figure 2B). This finding is in agreement with previous studies reporting exosome-rich pleural fluid derived from MPM patients [71,72], as well as a study conducted by Creaney et al., which established that MPM cells secrete higher levels of exosome-related proteins compared to non-malignant mesothelial cells [18]. Furthermore, our study revealed YM155-resistant MSTO-211H cells exhibit a higher level of exosome production in comparison to the YM155-sensitive H226 cells and that there was an evident suppression of exosome production for both of these cell lines following treatment with the GW4869 exosome inhibitor (Figure 3B). This result is in agreement with findings of a previous study by Munson et al., which reported that MPM cell secretion of tumour suppressor miRNA-rich exosomes was associated with enhanced tumorigenic capacity. This was confirmed upon inhibiting the MPM cell-mediated secretion of miR-16-5p-rich exosomes, which subsequently reduced tumorigenic capacity of the MPM cells [73]. In our study however, the subsequent treatment of the MSTO-211H cells with an exosome inhibitor did not sensitise them to YM155 treatment, therefore suggesting that MPM exosome production does not play a direct role in survivin-related small molecule inhibitor resistance in MPM.

To determine the mechanism associated with variable MPM cell drug response, as established from our in vitro and in vivo results for MSTO-211H and H226, we investigated the expression levels of a number of genes known to mediate anti-apoptosis (i.e., IAP gene family) and survivin-related drug uptake/transporter genes that are known to mediate drug resistance in other cancer types [74,75,76]. Out of all tested genes, there was no evident association between their expression and MPM cell response to YM155 small molecule inhibitor treatment (Figure 4A); with the exception of the drug transporter genes, *ABCA6* and *ABCA10*. Both of these genes are members of the ABC transporter gene family, which are known to contribute to chemotherapy drug resistance in cancer [77,78,79]. Our results indicated that both of these genes are over-expressed in a majority of the MPM cell lines in comparison to non-malignant mesothelial cells (Figure 4B). Furthermore, we showed that the expression of *ABCA6* and *ABCA10* is associated with poor MPM cell response to YM155 small molecule inhibitor treatment and silencing of these genes re-sensitises MPM cells to YM155 small molecule inhibitor treatment (Figure 4C,D), thus indicating a correlation between survivin expression and the *ABCA6/ABCA10* genes and their potential role in MPM drug response.

Collectively, the results from our study reinforce our understanding that chemotherapy and small molecule inhibitor drug response in MPM is complex and multi-faceted. There is an evident correlation between survivin expression and MPM drug response, however exosome production in MPM does not appear to play a direct role in survivin-mediated drug resistance. Rather, our study indicates that there is a link between the ABC drug transporter family and MPM response to survivin small molecule inhibitor treatment, which warrants further exploration in future preclinical studies. Furthermore, our study shows that restoration of tumour suppressor miRNA is able to enhance MPM cell response to chemotherapy drug and small molecule inhibitor treatment. Hence, prospective investigations aiming to explore combinational miRNA replacement with chemotherapy drug and/or small molecule inhibitors as a potential treatment option for MPM is greatly warranted.

## 5. Conclusions

This study demonstrated that the combination of small molecule inhibitor targeting agents of survivin and FAK with tumour-suppressor miRNA mimics, constitute promising efficacy as potential combinational treatment options for MPM that warrants further investigation. Upon exploring the potential mechanism responsible for variable MPM cell sensitivity to the survivin small molecule inhibitor, YM155, it was established that exosome involvement does not appear to be a contributing factor despite a high level of exosome secretion for most of the tested MPM cell lines. Furthermore, there was no evident correlation between survivin-related gene expression and MPM response to YM155. Rather, we determined that there is a potential link between the over-expression of the drug transporter genes, *ABCA6* and *ABCA10,* and reduced MPM sensitivity to YM155.

## Figures and Tables

**Table 1:** IC_50_ values and fold change increase in MPM cell sensitivity to chemotherapy/small molecule inhibitor drug treatment following miRNA transfection pre-treatment of MPM cells.

MicroRNA	PND-1186		YM155		Cisplatin		Gemcitabine	
	IC_50_ (µM)	Fold Change in Sensitivity	IC_50_ (nM)	Fold Change in Sensitivity	IC_50_ (µM)	Fold Change in Sensitivity	IC_50_ (µM)	Fold Change in Sensitivity
Control mimic	1.06 ± 0.60	-	117.10 ± 29.67	-	4.15 ± 0.35	-	0.24 ± 0.04	-
miR-17-5p	0.43 ± 0.02	2.47	-	-	-	-	-	-
miR-221-3p	0.20 ± 0.05	5.30	-	-	-	-	-	-
miR-222-3p	0.09 ± 0.03	11.78	89.36 ± 5.26	1.31	-	-	-	-
miR-137-3p	1.06 ± 0.30	1.00	171.75 ± 2.55	0.68	-	-	-	-
miR-148a-3p	0.83 ± 0.22	1.28	18.50 ± 4.68	6.33	-	-	-	-
miR-193a-3p	0.11 ± 0.06	9.64	20.97 ± 5.29	5.58	-	-	-	-
miR-142-5p	-	-	137.84 ± 4.91	0.85	-	-	-	-
miR-192-5p	-	-	67.94 ± 7.76	1.72	-	-	-	-
miR-122-5p	-	-	131.18 ± 3.88	0.89	-	-	-	-
miR-214-3p	-	-	76.73 ± 9.62	1.53	-	-	-	-
miR-486-5p	-	-	-	-	3.63 ± 0.48	1.18	0.26 ± 0.01	0.92
miR-31-3p	-	-	-	-	3.07 ± 0.25	1.39	0.26 ± 0.03	0.92
miR-31-5p	-	-	-	-	3.92 ± 0.12	1.09	0.28 ± 0.03	0.86
miR-145-3p	-	-	-	-	1.35 ± 0.14	3.16	0.21 ± 0.05	1.14
miR-145-5p	-	-	-	-	3.29 ± 0.11	1.30	0.25 ± 0.01	0.96
miR-15a-5p	-	-	-	-	1.43 ± 0.20	2.99	0.15 ± 0.02	1.60
miR-16-5p	-	-	-	-	0.86 ± 0.18	4.79	0.09 ± 0.0005	2.67
miR-34a-5p	-	-	-	-	3.33 ± 0.13	1.28	0.12 ± 0.05	2.00
miR-34b-3p	-	-	-	-	1.37 ± 0.22	3.12	0.11 ± 0.01	2.18

Note—IC_50_ values presented in the table were derived by taking the average of three IC_50_ values determined from experimental triplicates and are reported as IC_50_ value ± SD. MiRNAs that were not tested for each drug (i.e., no IC_50_ value was obtained) are indicated with (-). Fold change in sensitivity was determined with respect to the miRNA control mimic for each tested drug.

## Data Availability

The data presented in this study are available in this article (and supplementary material).

## Supplementary Materials

The following supporting information can be downloaded at: https://www.mdpi.com/article/10.3390/cancers14194784/s1, Figure S1: MicroRNAs associated with the survivin pathway; Figure S2: R code text file of drug IC_50_ calculations and statistical significance measures; Figure S3: MicroRNA upregulation in the MSTO-211H and VMC23 cell lines following miRNA mimic transfection; Figure S4: Reduction in expression of the *ABCA6* and *ABCA10* genes following siRNA treatment of the MSTO-211H cell line; Table S1: List of miRNAs and their corresponding sequences used for transfection of MPM cells; Table S2: List of siRNAs and their corresponding sequences used for transfection of VMC23 MPM cells; Table S3: List of primers used for RT-qPCR; Table S4: Statistical significance evaluation of observed increases in MPM cell sensitivity to chemotherapy/small molecule inhibitor drug treatment following miRNA pre-treatment of MPM cells; Table S5: Summary of MPM cell line mRNA expression levels corresponding to genes known to play a role in chemotherapy drug resistance; Table S6: Statistical significance evaluation of upregulated/downregulated gene expression of IAP, drug transporter and survivin-related genes; Table S7: Statistical significance evaluation of observed increases in MPM sensitivity to YM155 treatment of MSTO-211H MPM cells pre-treated with siRNA.

## References

[1] Zalcman G., Mazieres J., Margery J., Greillier L., Audigier-Valette C., Moro-Sibilot D., Molinier O., Corre R., Monnet I., Gounant V. (2016). Bevacizumab for newly diagnosed pleural mesothelioma in the Mesothelioma Avastin Cisplatin Pemetrexed Study (MAPS): A randomised, controlled, open-label, phase 3 trial. Lancet.

[2] Vogelzang N.J., Rusthoven J.J., Symanowski J., Denham C., Kaukel E., Ruffie P., Gatzemeier U., Boyer M., Emri S., Manegold C. (2003). Phase III study of pemetrexed in combination with cisplatin versus cisplatin alone in patients with malignant pleural mesothelioma. J. Clin. Oncol..

[3] Tsao A.S., Wistuba I., Roth J.A., Kindler H.L. (2009). Malignant pleural mesothelioma. J. Clin. Oncol..

[4] Hudson A.L., Weir C., Moon E., Harvie R., Klebe S., Clarke S., Pavlakis N., Howell V.M. (2014). Establishing a panel of chemo-resistant mesothelioma models for investigating chemo-resistance and identifying new treatments for mesothelioma. Sci. Rep..

[5] Moody H., Lind M.J., Maher S.G. (2017). MicroRNA-31 Regulates Chemosensitivity in Malignant Pleural Mesothelioma. Mol. Ther.—Nucleic Acids.

[6] Meads M.B., Gatenby R.A., Dalton W.S. (2009). Environment-mediated drug resistance: A major contributor to minimal residual disease. Nat. Cancer.

[7] Hazlehurst L.A., Dalton W.S. (2001). Mechanisms associated with cell adhesion mediated drug resistance (CAM-DR) in hematopoietic malignancies. Cancer Metastasis Rev..

[8] Guan J., Chen J. (2013). Mesenchymal stem cells in the tumor microenvironment. Biomed. Rep..

[9] Valadi H., Ekström K., Bossios A., Sjöstrand M., Lee J.J., Lötvall J.O. (2007). Exosome-mediated transfer of mRNAs and microRNAs is a novel mechanism of genetic exchange between cells. Nat. Cell Biol..

[10] Steinbichler T.B., Dudás J., Skvortsov S., Ganswindt U., Riechelmann H., Skvortsova I.I. (2019). Therapy resistance mediated by exosomes. Mol. Cancer.

[11] Camussi G., Deregibus M.-C., Bruno S., Grange C., Fonsato V., Tetta C. (2010). Exosome/microvesicle-mediated epigenetic reprogramming of cells. Am. J. Cancer Res..

[12] Costa-Silva B., Aiello N.M., Ocean A.J., Singh S., Zhang H., Thakur B.K., Becker A., Hoshino A., Mark M.T., Molina H. (2015). Pancreatic cancer exosomes initiate pre-metastatic niche formation in the liver. Nat. Cell Biol..

[13] Peinado H., Alečković M., Lavotshkin S., Matei I., Costa-Silva B., Moreno-Bueno G., Hergueta-Redondo M., Williams C., García-Santos G., Ghajar C.M. (2012). Melanoma exosomes educate bone marrow progenitor cells toward a pro-metastatic phenotype through MET. Nat. Med..

[14] Vaksman O., Tropé C., Davidson B., Reich R. (2014). Exosome-derived miRNAs and ovarian carcinoma progression. Carcinogenesis.

[15] Ono M., Kosaka N., Tominaga N., Yoshioka Y., Takeshita F., Takahashi R.-U., Yoshida M., Tsuda H., Tamura K., Ochiya T. (2014). Exosomes from bone marrow mesenchymal stem cells contain a microRNA that promotes dormancy in metastatic breast cancer cells. Sci. Signal..

[16] Greening D.W., Ji H., Chen M., Robinson B.W.S., Dick I.M., Creaney J., Simpson R.J. (2016). Secreted primary human malignant mesothelioma exosome signature reflects oncogenic cargo. Sci. Rep..

[17] Hegmans J.P., Bard M.P., Hemmes A., Luider T.M., Kleijmeer M.J., Prins J.-B., Zitvogel L., Burgers S.A., Hoogsteden H.C., Lambrecht B.N. (2004). Proteomic analysis of exosomes secreted by human mesothelioma cells. Am. J. Pathol..

[18] Creaney J., Dick I.M., Leon J.S., Robinson B.W. (2017). A Proteomic Analysis of the Malignant Mesothelioma Secretome Using iTRAQ. Cancer Genom.—Proteom..

[19] Zhong Y., Li H., Li P., Chen Y., Zhang M., Yuan Z., Zhang Y., Xu Z., Luo G., Fang Y. (2021). Exosomes: A New Pathway for Cancer Drug Resistance. Front. Oncol..

[20] Dong X., Bai X., Ni J., Zhang H., Duan W., Graham P., Li Y. (2020). Exosomes and breast cancer drug resistance. Cell Death Dis..

[21] Mita A.C., Mita M.M., Nawrocki S.T., Giles F.J. (2008). Survivin: Key Regulator of Mitosis and Apoptosis and Novel Target for Cancer Therapeutics. Clin. Cancer Res..

[22] Schimmer A.D. (2004). Inhibitor of Apoptosis Proteins: Translating Basic Knowledge into Clinical Practice. Cancer Res..

[23] Hmeljak J., Erčulj N., Dolžan V., Kern I., Cör A. (2011). BIRC5 promoter SNPs do not affect nuclear survivin expression and survival of malignant pleural mesothelioma patients. J. Cancer Res. Clin. Oncol..

[24] Falleni M., Pellegrini C., Marchetti A., Roncalli M., Nosotti M., Palleschi A., Santambrogio L., Coggi G., Bosari S. (2005). Quantitative evaluation of the apoptosis regulating genes Survivin, Bcl-2 and Bax in inflammatory and malignant pleural lesions. Lung Cancer.

[25] Gordon G., Mani M., Mukhopadhyay L., Dong L., Edenfield H., Glickman J., Yeap B., Sugarbaker D., Bueno R. (2007). Expression patterns of inhibitor of apoptosis proteins in malignant pleural mesothelioma. J. Pathol..

[26] Zaffaroni N., Costa A., Pennati M., De Marco C., Affini E., Madeo M., Erdas R., Cabras A.D., Kusamura S., Baratti D. (2007). Survivin is highly expressed and promotes cell survival in malignant peritoneal mesothelioma. Cell. Oncol..

[27] Kleinberg L., Lie A.K., Flørenes V.A., Nesland J.M., Davidson B. (2007). Expression of inhibitor-of-apoptosis protein family members in malignant mesothelioma. Hum. Pathol..

[28] Als A.B., Dyrskjøt L., von der Maase H., Koed K., Mansilla F., Toldbod H.E., Jensen J.L., Ulhøi B.P., Sengeløv L., Jensen K.M. (2007). Emmprin and Survivin Predict Response and Survival following Cisplatin-Containing Chemotherapy in Patients with Advanced Bladder Cancer. Clin. Cancer Res..

[29] Xia C., Xu Z., Yuan X., Uematsu K., You L., Li K., Li L., McCormick F., Jablons D.M. (2002). Induction of apoptosis in mesothelioma cells by antisurvivin oligonucleotides. Mol. Cancer Ther..

[30] Gordon G., Mani M., Mukhopadhyay L., Dong L., Yeap B., Sugarbaker D., Bueno R. (2007). Inhibitor of apoptosis proteins are regulated by tumour necrosis factor-α in malignant pleural mesothelioma. J. Pathol..

[31] Kim K.W., Mutter R.W., Willey C.D., Subhawong T.K., Shinohara E.T., Albert J.M., Ling G., Cao C., Gi Y.J., Lu B. (2007). Inhibition of survivin and aurora B kinase sensitizes mesothelioma cells by enhancing mitotic arrests. Int. J. Radiat. Oncol..

[32] Zaffaroni N., Daidone M.G. (2002). Survivin expression and resistance to anticancer treatments: Perspectives for new therapeutic interventions. Drug Resist. Updat..

[33] Goričar K., Kovač V., Franko A., Dodič-Fikfak M., Dolžan V. (2015). Serum Survivin Levels and Outcome of Chemotherapy in Patients with Malignant Mesothelioma. Dis. Markers.

[34] Williams M., Cheng Y.Y., Blenkiron C., Reid G. (2017). Exploring Mechanisms of MicroRNA Downregulation in Cancer. Microrna.

[35] Blower P.E., Verducci J.S., Lin S., Zhou J., Chung J.-H., Dai Z., Liu C.-G., Reinhold W., Lorenzi P.L., Kaldjian E.P. (2007). MicroRNA expression profiles for the NCI-60 cancer cell panel. Mol. Cancer Ther..

[36] Williams M., Cheng Y.Y., Phimmachanh M., Winata P., Van Zandwijk N., Reid G. (2019). Tumour suppressor microRNAs contribute to drug resistance in malignant pleural mesothelioma by targeting anti-apoptotic pathways. Cancer Drug Resist..

[37] Reid G., Pel M.E., Kirschner M.B., Cheng Y.Y., Mugridge N., Weiss J., Williams M., Wright C., Edelman J.J.B., Vallely M.P. (2013). Restoring expression of miR-16: A novel approach to therapy for malignant pleural mesothelioma. Ann. Oncol..

[38] Kao S.C., Fulham M., Wong K., Cooper W., Brahmbhatt H., MacDiarmid J., Pattison S., Sagong J.O., Huynh Y., Leslie F. (2015). A Significant Metabolic and Radiological Response after a Novel Targeted MicroRNA-based Treatment Approach in Malignant Pleural Mesothelioma. Am. J. Respir. Crit. Care Med..

[39] van Zandwijk N., Pavlakis N., Kao S.C., Linton A., Boyer M.J., Clarke S., Huynh Y., Chrzanowska A., Fulham M.J., Bailey D.L. (2017). Safety and activity of microRNA-loaded minicells in patients with recurrent malignant pleural mesothelioma: A first-in-man, phase 1, open-label, dose-escalation study. Lancet Oncol..

[40] Yuen M.L., Zhuang L., Rath E.M., Yu T., Johnson B., Sarun K.H., Wang Y., Kao S., Linton A., Clarke C.J. (2021). The Role of E-Cadherin and microRNA on FAK Inhibitor Response in Malignant Pleural Mesothelioma (MPM). Int. J. Mol. Sci..

[41] Schedlich L.J., Cheng Y.Y., Gattani S., Cheng N.C., Kirschner M.B., van Zandwijk N., Reid G. The survivin suppressant YM 155 selectively inhibits the growth of epithelioid malignant mesothelioma in vitro and in vivo. Proceedings of the 11th International Mesothelioma Interest Group (iMig).

[42] Pinelli S., Alinovi R., Poli D., Corradi M., Pelosi G., Tiseo M., Goldoni M., Cavallo D., Mozzoni P. (2021). Overexpression of microRNA-486 affects the proliferation and chemosensitivity of mesothelioma cell lines by targeting *PIM1*. Int. J. Mol. Med..

[43] Smythe W., Kaiser L.R., Hwang H.C., Amin K.M., Pilewski J.M., Eck S.J., Wilson J.M., Albelda S.M. (1994). Successful adenovirus-mediated gene transfer in an in vivo model of human malignant mesothelioma. Ann. Thorac. Surg..

[44] Kao S.C., Cheng Y.Y., Williams M., Kirschner M.B., Madore J., Lum T., Sarun K.H., Linton A., McCaughan B., Klebe S. (2017). Tumor Suppressor microRNAs Contribute to the Regulation of PD-L1 Expression in Malignant Pleural Mesothelioma. J. Thorac. Oncol..

[45] Cheng Y.Y., Kirschner M.B., Cheng N.C., Gattani S., Klebe S., Edelman J.J.B., Vallely M.P., McCaughan B.C., Jin H.C., van Zandwijk N. (2013). ZIC1 Is silenced and has tumor suppressor function in malignant pleural mesothelioma. J. Thorac. Oncol..

[46] Rath E.M., Cheng Y.Y., Pinese M., Sarun K.H., Hudson A.L., Weir C., Wang Y.D., Håkansson A.P., Howell V.M., Liu G.-J. (2018). BAMLET kills chemotherapy-resistant mesothelioma cells, holding oleic acid in an activated cytotoxic state. PLoS ONE.

[47] von Seggern D.H. (2007). CRC Standard Curves and Surfaces with Mathematica.

[48] Nash C.J., Varadhan R. (2011). Unifying Optimization Algorithms to Aid Software System Users: Optimx for R. J. Stat. Softw..

[49] R Core Team (2020). R: A Language and Environment for Statistical Computing.

[50] Yasuhisa K., Morita S., Matsuo M., Ueda K. (2007). Mechanism of multidrug recognition by MDR1/ABCB1. Cancer Sci..

[51] Park E., Gang E.J., Hsieh Y.-T., Schaefer P., Chae S., Klemm L., Huantes S., Loh M., Conway E., Kang E.-S. (2011). Targeting survivin overcomes drug resistance in acute lymphoblastic leukemia. Blood.

[52] Zhang Y., Wang J. (2017). MicroRNAs are important regulators of drug resistance in colorectal cancer. Biol. Chem..

[53] Tanaka N., Toyooka S., Soh J., Tsukuda K., Shien K., Furukawa M., Muraoka T., Maki Y., Ueno T., Yamamoto H. (2013). Downregulation of microRNA-34 induces cell proliferation and invasion of human mesothelial cells. Oncol. Rep..

[54] Andersen M., Trapani D., Ravn J., Sørensen J.B., Andersen C.B., Grauslund M., Santoni-Rugiu E. (2015). Methylation-associated Silencing of microRNA-126 and its Host Gene EGFL7 in Malignant Pleural Mesothelioma. Anticancer Res..

[55] Lin H.-M., Nikolic I., Yang J., Castillo L., Deng N., Chan C.-L., Yeung N.K., Dodson E., Elsworth B., Spielman C. (2018). MicroRNAs as potential therapeutics to enhance chemosensitivity in advanced prostate cancer. Sci. Rep..

[56] Mognato M., Celotti L. (2015). MicroRNAs Used in Combination with Anti-Cancer Treatments Can Enhance Therapy Efficacy. Mini-Rev. Med. Chem..

[57] Shanthi E., Krishna M.H., Arunesh G.M., Reddy K.V., Kumar J.S., Viswanadhan V.N. (2014). Focal adhesion kinase inhibitors in the treatment of metastatic cancer: A patent review. Expert Opin. Ther. Patents.

[58] Golubovskaya V.M., Cance W.G. (2007). Focal adhesion kinase and p53 signaling in cancer cells. Int. Rev. Cytol..

[59] Moen I., Gebre M., Alonso-Camino V., Chen D., Epstein D., McDonald D.M. (2015). Anti-metastatic action of FAK inhibitor OXA-11 in combination with VEGFR-2 signaling blockade in pancreatic neuroendocrine tumors. Clin. Exp. Metastasis.

[60] Jiang H., Hegde S., Knolhoff B.L., Zhu Y., Herndon J.M., Meyer M.A., Nywening T.M., Hawkins W.G., Shapiro I.M., Weaver D.T. (2016). Targeting focal adhesion kinase renders pancreatic cancers responsive to checkpoint immunotherapy. Nat. Med..

[61] Soria J.C., Gan H.K., Blagden S.P., Plummer R., Arkenau H.T., Ranson M., Evans T.R.J., Zalcman G., Bahleda R., Hollebecque A. (2016). A phase I, pharmacokinetic and pharmacodynamic study of GSK2256098, a focal adhesion kinase inhibitor, in patients with advanced solid tumors. Ann. Oncol..

[62] Mak G., Soria J.-C., Blagden S.P., Plummer R., Fleming R.A., Nebot N., Zhang J., Mazumdar J., Rogan D., Gazzah A. (2019). A phase Ib dose-finding, pharmacokinetic study of the focal adhesion kinase inhibitor GSK2256098 and trametinib in patients with advanced solid tumours. Br. J. Cancer.

[63] Cheng X.J., Lin J.C., Ding Y.F., Zhu L., Ye J., Tu S.P. (2016). Survivin inhibitor YM155 suppresses gastric cancer xenograft growth in mice without affecting normal tissues. Oncotarget.

[64] Nakahara T., Takeuchi M., Kinoyama I., Minematsu T., Shirasuna K., Matsuhisa A., Kita A., Tominaga F., Yamanaka K., Kudoh M. (2007). YM155, a Novel Small-Molecule Survivin Suppressant, Induces Regression of Established Human Hormone-Refractory Prostate Tumor Xenografts. Cancer Res..

[65] Kumar B., Yadav A., Lang J.C., Cipolla M.J., Schmitt A.C., Arradaza N., Teknos T.N., Kumar P. (2012). YM155 reverses cisplatin resistance in head and neck cancer by decreasing cytoplasmic survivin levels. Mol. Cancer Ther..

[66] Huang J., Lyu H., Wang J., Liu B. (2014). MicroRNA regulation and therapeutic targeting of survivin in cancer. Am. J. Cancer Res..

[67] Russo G.L., Tessari A., Capece M., Galli G., De Braud F., Garassino M.C., Palmieri D. (2018). MicroRNAs for the Diagnosis and Management of Malignant Pleural Mesothelioma: A Literature Review. Front. Oncol..

[68] Wang B., Li X., Zhao G., Yan H., Dong P., Watari H., Sims M., Li W., Pfeffer L.M., Guo Y. (2018). miR-203 inhibits ovarian tumor metastasis by targeting BIRC5 and attenuating the TGFβ pathway. J. Exp. Clin. Cancer Res..

[69] Ferrario A., Luna M., Rucker N., Wong S., Lederman A., Kim J., Gomer C. (2016). Targeting Survivin Enhances Chemosensitivity in Retinoblastoma Cells and Orthotopic Tumors. PLoS ONE.

[70] Kelly R.J., Thomas A., Rajan A., Chun G., Lopez-Chavez A., Szabo E., Spencer S., Carter C.A., Guha U., Khozin S. (2013). A phase I/II study of sepantronium bromide (YM155, survivin suppressor) with paclitaxel and carboplatin in patients with advanced non-small-cell lung cancer. Ann. Oncol..

[71] Bard M.P., Hegmans J.P., Hemmes A., Luider T.M., Willemsen R., Severijnen L.-A.A., van Meerbeeck J.P., Burgers S.A., Hoogsteden H.C., Lambrecht B.N. (2004). Proteomic Analysis of Exosomes Isolated from Human Malignant Pleural Effusions. Am. J. Respir. Cell Mol. Biol..

[72] Mahaweni N.M., Kaijen-Lambers M.E., Dekkers J., Aerts J.G., Hegmans J.P. (2013). Tumour-derived exosomes as antigen delivery carriers in dendritic cell-based immunotherapy for malignant mesothelioma. J. Extracell. Vesicles.

[73] Munson P.B., Hall E.M., Farina N.H., Pass H., Shukla A. (2019). Exosomal miR-16-5p as a target for malignant mesothelioma. Sci. Rep..

[74] Rathore R., McCallum J.E., Varghese E., Florea A.-M., Büsselberg D. (2017). Overcoming chemotherapy drug resistance by targeting inhibitors of apoptosis proteins (IAPs). Apoptosis Int. J. Program. Cell Death.

[75] Tamm I., Kornblau S.M., Segall H., Krajewski S., Welsh K., Kitada S., Scudiero D.A., Tudor G., Qui Y.H., Monks A. (2000). Expression and Prognostic Significance of IAP-Family Genes in Human Cancers and Myeloid Leukemias. Clin. Cancer Res..

[76] Choi H.Y., Yu A.-M. (2014). ABC transporters in multidrug resistance and pharmacokinetics, and strategies for drug development. Curr. Pharm. Des..

[77] Pasello M., Giudice A.M., Scotlandi K. (2020). The ABC subfamily A transporters: Multifaceted players with incipient potentialities in cancer. Semin. Cancer Biol..

[78] Albrecht C., Viturro E. (2007). The ABCA subfamily—Gene and protein structures, functions and associated hereditary diseases. Pflügers Arch.—Eur. J. Physiol..

[79] Gillet J.-P., Efferth T., Steinbach D., Hamels J., de Longueville F., Bertholet V., Remacle J. (2004). Microarray-based Detection of Multidrug Resistance in Human Tumor Cells by Expression Profiling of ATP-binding Cassette Transporter Genes. Cancer Res..

