# Supplementary material for: Exploring MicroRNA and Exosome Involvement in Malignant Pleural Mesothelioma Drug Response
Source: Cancers (Basel). 2022 Sep 30;14(19):4784. doi: 10.3390/cancers14194784 (PMC9564288; doi:10.3390/cancers14194784)
Supplement: Supplementary file 1 [file cancers-14-04784-s001.zip › cancers-1950371-supplementary Figures S1, S3, S4 and Tables S1-S7.pdf]

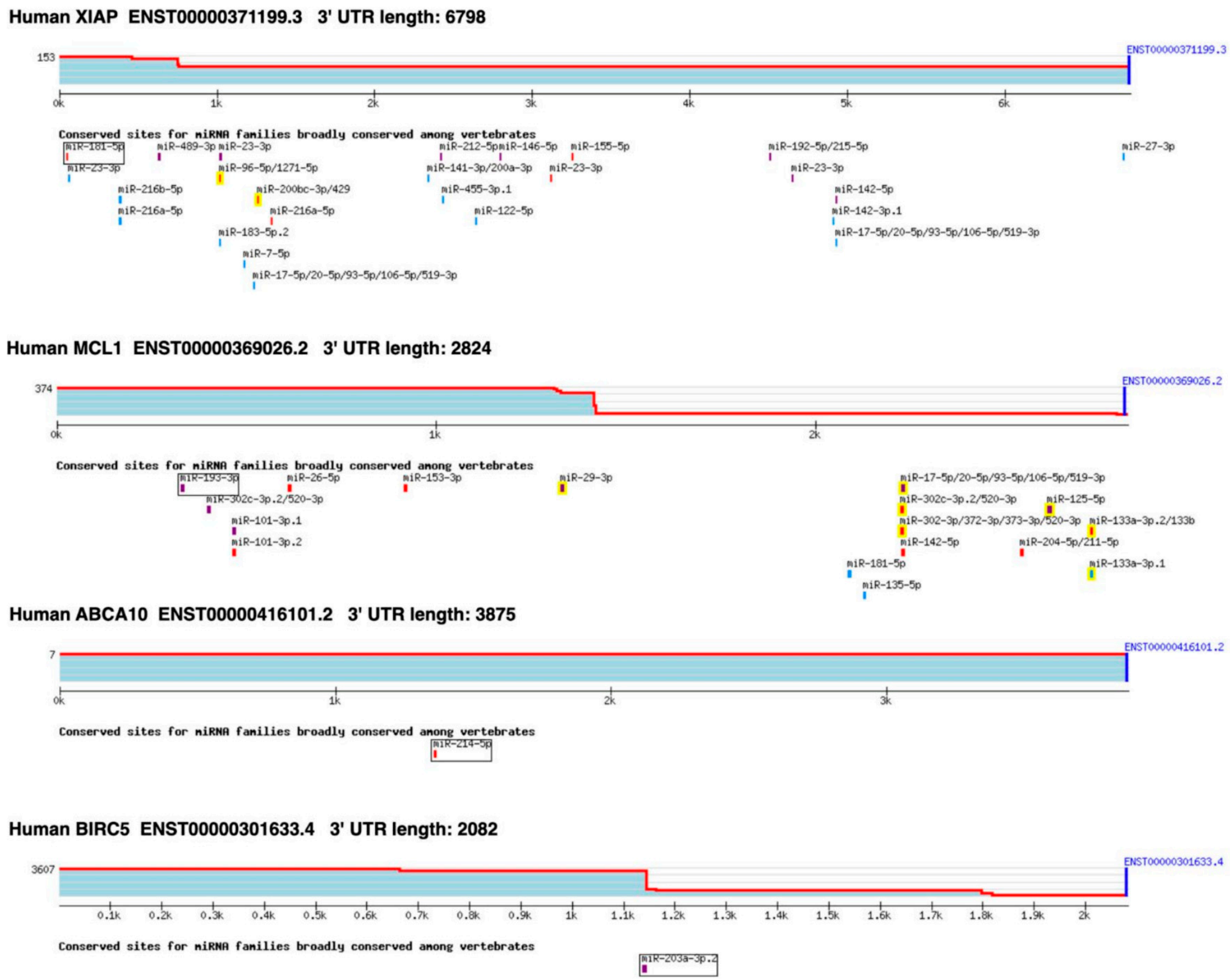

Figure S1. MicroRNAs associated with the survivin pathway. Target scan results depict miRNAs that correspond to human XIAP, MCL1, ABCA10 and BIRC5.

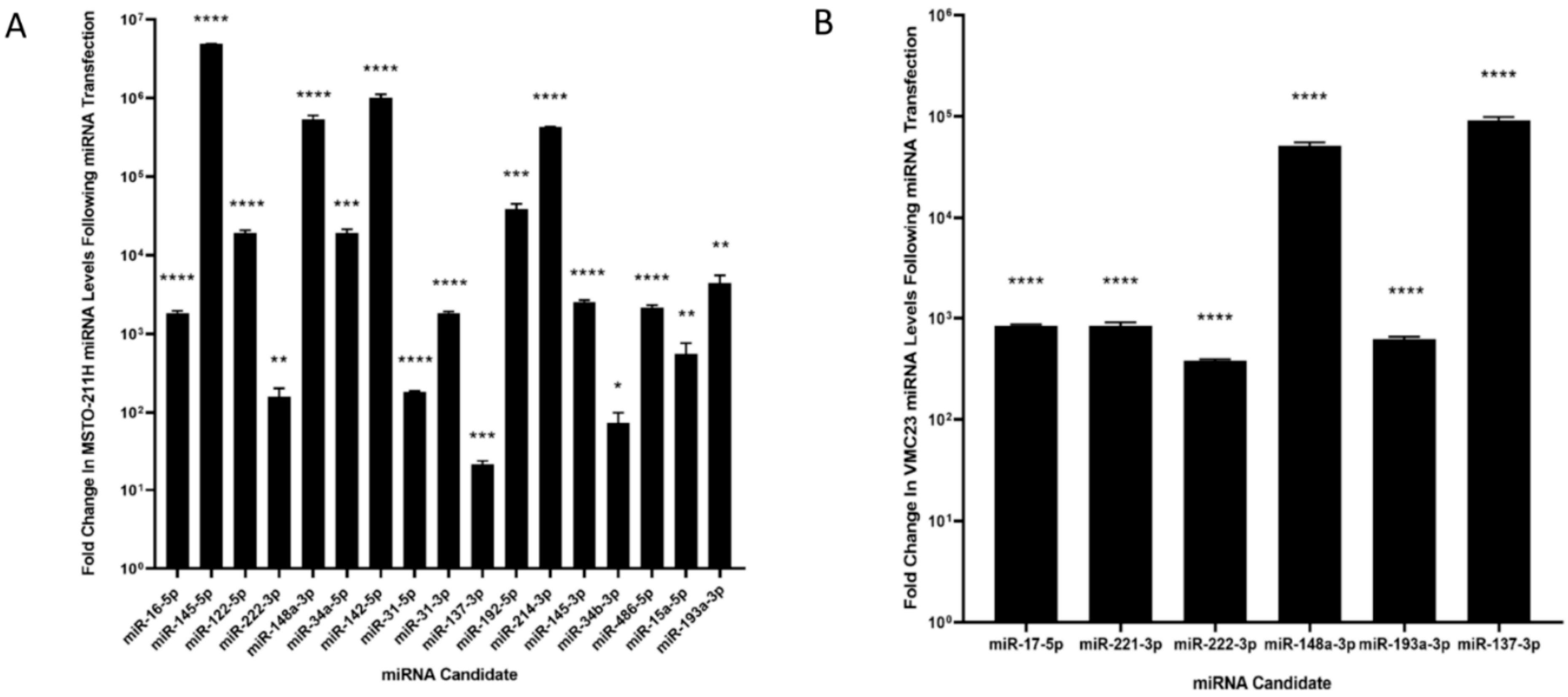

Figure S3. MicroRNA upregulation in the MSTO-211H and VMC23 cell lines following miRNA mimic transfection. Graphs depicting the fold change in miRNA expression in (A) MSTO-211H and (B) VMC23 cells following 24 h of 1 nM miRNA transfection. The post-transfection miRNA levels were quantified with respect to the miRNA levels of the untransfected control cells (normalised to a value of 1 but not shown on the graphs). Error bars represent the mean ± SD, as determined from three experimental replicates. Statistically significant fold changes in miRNA expression were determined via a Student's t-test, whereby a pvalue of ≤ 0.05, ≤ 0.005, ≤ 0.0005 and ≤ 0.00005 is indicated on the graphs as *, **, ***, and ****, respectively.

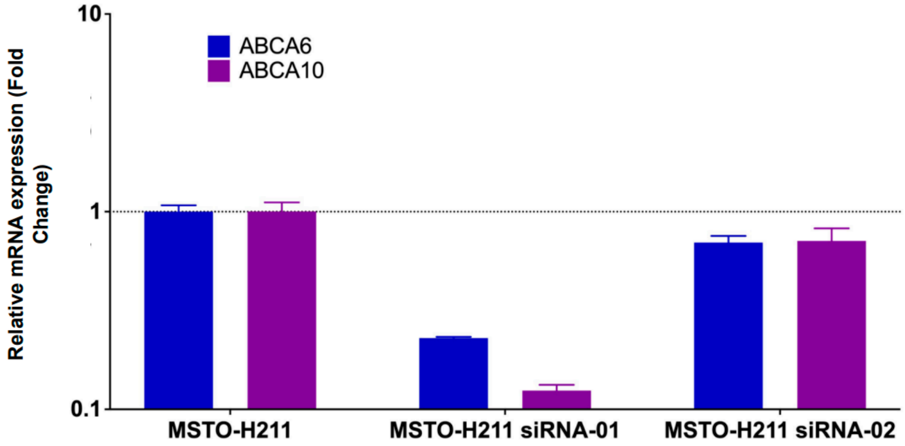

Figure S4. Reduction in expression of the ABCA6 and ABCA10 genes following siRNA treatment of the MSTO-211H cell line. The ABCA6 and ABCA10 mRNA levels of the untreated MSTO-211H control cells were normalised to a value of 1, as indicated by the horizontal line on the graph.

Table S1. List of miRNAs and their corresponding sequences used for transfection of MPM cells.

miRNA name	Sequence 5' to 3'	TaqMan Assay ID
control mimic	UUCUCCGAACGUGUCACGUTT	N/A
miR-486-5p	UCCUGUACUGAGCUGCCCCGAG	001278
miR-31-5p	AGGCAAGAUGCUGGCAUAGCU	002279
miR-31-3p	UGCUAUGCCAACAUAUUGCCAU	002113
miR-145-3p	GGAUUCCUGGAAAUACUGUUCU	002149
miR-145-5p	GUCCAGUUUUCCCAGGAAUCCCU	002278
miR-15a-5p	UAGCAGCACAUAAUGGUUUGUG	000389
miR-16-5p	UAGCAGCACGUAAAUAUUGGCG	000391
miR-34a-5p	UGGCAGUGUCUUAGCUGGUUGU	000426
miR-34b-3p	CAAUCAGCAAGUAUACUGCCCU	002102
miR-137-3p	UUAUUGCUUAAGAAUACGCGUAG	001129
miR-142-5p	CAUAAAGUAGAAAGCACUACU	002248
miR-193a-3p	AACUGGCCUACAAAGUCCCAGU	002250
miR-192-5p	CUGACCUAUGAAUUGACAGCC	000491
miR-122-5p	UGGAGUGUGACAAUGGUGUUUG	002245
miR-222-3p	AGCUACAUCUGGCUACUGGGU	002276
miR-214-3p	ACAGCAGGCACAGACAGGCAGU	002306
miR-148a-3p	UCAGUGCACUACAGAACUUUGU	000470
miR-17-5p	CAAAGUGCUUACAGUGCAGGUAG	000393
miR-221-3p	AGCUACAUUGUCUGCUGGGUUUC	000524

Note - The corresponding TaqMan assay ID's for the miRNA RT-qPCR quantification experiments are shown above.
RNU6B (TaqMan Assay ID 001093) was utilised as an endogenous reference for the RT-qPCR miRNA quanitification experiments.

Table S2. List of siRNAs and their corresponding sequences used for transfection of VMC23 MPM cells.

siRNA name	Sense strand sequence 5' to 3'	Antisense strand sequence 5' to 3'
si-ABCA6-01	GCAUCCCUCUGAUGAUUAUTT	AUAAUCAUCAGAGGGAUGCTT
si-ABCA6-02	GCAAUGGGCUACUUCAAAUTT	AUUUGAAGUAGCCCAUUGCTT
si-ABCA10-01	GCUUAUAUGGCCUUUCUUUTT	AAAGAAAGGCCAUAUAAGCTT
si-ABCA10-02	GCAAUGCCCUUAUGGGAAUTT	AUUCCCAUAAGGGCAUUGCTT

Table S3. List of primers used for RT-qPCR.

Primer name	Sequence 5' to 3'
ABCA6 F	TTGGAATTGGACATGCAAAA
ABCA6 R	TCTCAGGAGGCTCCACACTT
ABCA10 F	AGTGGCAACACTTCGCTTCT
ABCA10 R	CGGGATTTGTTCCAGAGAAA
OCT1 F	TAATGGACCACATCGCTCAA
OCT1 R	AGCCCCTGATAGAGCACAGA
ABCB1 F	GCTCCTGACTATGCCAAAGC
ABCB1 R	TCTTCACCTCCAGGCTCAGT
MRP1 F	AAGAAAACAGGGAAGCAGCA
MRP1 R	GCTCTCTGGGTTTGAAGTCG
BCRP F	GACGTGTCGGTGATGATGAG
BCRP R	ACCTTATTGACCGCCTCCTT
IAP1 F	TATAGGGCAGGGCCTGTATG
IAP1 R	CAGCCTCATTCTCCCTGAAG
IAP2 F	ATGCTGCAGGAAATGGAAAC
IAP2 R	CAACTTTTGGGGGAAGTCAA
ILF3 F	GGGCGGAGATTTCTACCTTC
ILF3 R	AGACACGGAGTCCCAAACAC
BIRC5 F	GGACCACCGCATCTCTACAT
BIRC5 R	GACAGAAAGGAAAGCGCAAC
ABCG2 F	CCTTCTTCTAGCCCCTTGCT
ABCG2 R	GAGGAAATGTCCCGAGTGAA
XIAP F	GGGGTTCAGTTTCAAGGACA
XIAP R	CGCCTTAGCTGCTCTTCAGT
PTK2 F	GCCTTATGACGAAATGCTGGGC
PTK2 R	CCTGTCTTCTGGACTCCATCCT

Note - F and R refer to forward and reverse primer, respectively.

Table S4. Statistical significance evaluation of observed increases in MPM cell sensitivity to chemotherapy / small molecule inhibitor drug treatment following miRNA pre-treatment of MPM cells.

Cell Line	Drug	miRNA	P-Value	Statistically Significant Increase In Sensitivity?
MSTO-211H	YM155	miR-122-5p	0.0014	Yes
MSTO-211H	YM155	miR-137-3p	1.0000	No
MSTO-211H	YM155	miR-142-5p	0.0226	Yes
MSTO-211H	YM155	miR-148a-3p	1.0411E-27	Yes
MSTO-211H	YM155	miR-192-5p	5.5834E-14	Yes
MSTO-211H	YM155	miR-193a-3p	8.9741E-17	Yes
MSTO-211H	YM155	miR-214-3p	1.0682E-08	Yes
MSTO-211H	YM155	miR-222-3p	6.4176E-10	Yes
MSTO-211H	Cisplatin	miR-145-3p	2.1311E-21	Yes
MSTO-211H	Cisplatin	miR-145-5p	6.2134E-11	Yes
MSTO-211H	Cisplatin	miR-15a-5p	1.2592E-19	Yes
MSTO-211H	Cisplatin	miR-16-5p	1.2854E-22	Yes
MSTO-211H	Cisplatin	miR-31-3p	2.3561E-09	Yes
MSTO-211H	Cisplatin	miR-31-5p	0.0038	Yes
MSTO-211H	Cisplatin	miR-34a-5p	4.0095E-08	Yes
MSTO-211H	Cisplatin	miR-34b-3p	5.9751E-17	Yes
MSTO-211H	Cisplatin	miR-486-5p	2.1311E-21	Yes
MSTO-211H	Gemcitabine	miR-145-3p	0.0151	Yes
MSTO-211H	Gemcitabine	miR-145-5p	1.0000	No
MSTO-211H	Gemcitabine	miR-15a-5p	9.9759E-06	Yes
MSTO-211H	Gemcitabine	miR-16-5p	2.0807E-17	Yes
MSTO-211H	Gemcitabine	miR-31-3p	0.3975	No
MSTO-211H	Gemcitabine	miR-31-5p	0.0090	No
MSTO-211H	Gemcitabine	miR-34a-5p	7.0774E-05	Yes
MSTO-211H	Gemcitabine	miR-34b-3p	6.9746E-10	Yes
MSTO-211H	Gemcitabine	miR-486-5p	0.3275	No
VMC23	PND-1186	miR-17-5p	0.0002	Yes
VMC23	PND-1186	miR-221-3p	2.2676E-08	Yes
VMC23	PND-1186	miR-222-3p	2.1908E-08	Yes
VMC23	PND-1186	miR-137-3p	0.7465	No
VMC23	PND-1186	miR-148a-3p	0.3963	No
VMC23	PND-1186	miR-193a-3p	1.8627E-09	Yes

Note - Statistical significance results presented in the table were determined by measuring the p-value of chi-squared statistical analyses comparing nested sigmoidal models of observed increases in MPM cell sensitivity to chemotherapy / small molecule inhibitor drug treatment of miRNA-treated MPM cells with respect to the inactive miRNA control mimic-treated cells. MiRNA and drug treatment combinations yielding a p-value of ≤ 0.05 (that were associated with a fold-change increase in MPM cell sensitivity) were deemed to induce a statistically significant increase in sensitivity.

Table S5. Summary of MPM cell line mRNA expression levels corresponding to genes known to play a role in chemotherapy drug resistance.

	H226		Ren		VMC40		VMC20		H2052		H2452		H28		MSTO		VMC23		MM05	
**Gene (mRNA)**	**Fold Change**	**P-Value**	**Fold Change**	**P-Value**	**Fold Change**	**P-Value**	**Fold Change**	**P-Value**	**Fold Change**	**P-Value**	**Fold Change**	**P-Value**	**Fold Change**	**P-Value**	**Fold Change**	**P-Value**	**Fold Change**	**P-Value**	**Fold Change**	**P-Value**
OCT1	**1.28**	**0.04371**	0.8143	0.25534	**2.9924**	**0.01075**	**9.8863**	**0.00040**	1.2598	0.41571	**5.9467**	**0.04493**	**0.2893**	**0.01335**	**0.6029**	**0.01001**	**0.4458**	**0.01990**	**14.0422**	**0.00138**
MDR1 (ABCB1)	**2060.6075**	**0.00601**	**59.8724**	**0.01060**	**13.7034**	**0.01479**	**11896.5600**	**0.01205**	**670.6964**	**0.00976**	50.3864	0.05318	**1343.2880**	**0.01416**	7560.1945	0.10641	**1354.5045**	**0.02424**	**5001.8400**	**0.02681**
MRP1	0.9863	0.22436	1.2079	0.12696	0.2394	0.06632	**5.5596**	**0.00559**	**1.8526**	**0.04376**	2.5299	0.07957	1.0178	0.19730	**2.8226**	**0.03641**	0.2644	0.06821	62.5710	0.06284
BCRP (ABCG2)	1.8315	0.07887	**3.5102**	**0.02604**	**0.0001**	**0.00038**	**62.2369**	**0.02553**	**0.3686**	**0.00103**	0.8096	0.22846	**0.0631**	**0.00045**	**2.0012**	**0.00260**	**0.1207**	**0.00089**	**6.8927**	**0.00008**
IAP1	1.7065	0.08215	**12.8679**	**0.00107**	0.8231	0.32558	**23.7682**	**0.01127**	**1.9438**	**0.04918**	1.4725	0.12237	0.5357	0.45042	2.8653	0.07329	1.1633	0.18401	**12.2211**	**0.02561**
IAP2	**37.8245**	**0.00091**	**202.5697**	**0.00499**	**88.1701**	**0.00156**	**503.2797**	**0.00015**	**329.7472**	**0.00015**	**51.5841**	**0.00621**	**30.0454**	**0.04257**	**161.7682**	**0.00534**	**128.4452**	**0.00001**	**263.8947**	**0.00929**
ILF3	1.2498	0.10017	**2.9070**	**0.02361**	0.6925	0.46303	**11.9322**	**0.00551**	0.3544	0.15230	0.3646	0.15453	0.2671	0.11437	0.9755	0.36258	0.2052	0.09859	**8.7546**	**0.00059**
Birc5	0.9149	0.12107	**2.5232**	**0.02611**	**1.2231**	**0.01114**	**5.6381**	**0.03174**	**0.4378**	**0.00785**	**0.3550**	**0.00139**	**0.3112**	**0.00711**	1.7544	0.08373	**0.2116**	**0.00082**	**4.2191**	**0.04640**
ABCG2	**1.5679**	**0.03300**	**3.4748**	**0.00415**	**0.2600**	**0.03537**	**794.2575**	**0.00238**	0.5854	0.13145	1.9100	0.07379	**0.0181**	**0.01816**	13.6587	0.05658	0.4664	0.07624	**21.0549**	**0.04166**
XIAP	1.6574	0.07824	**3.2738**	**0.02123**	**2.6991**	**0.02183**	**27.5540**	**0.00307**	**1.9879**	**0.01736**	1.0733	0.23948	0.5710	0.26741	5.0701	0.07084	1.0537	0.17020	22.8125	0.05393
ABCA6	**0.0710**	**0.01224**	0.5890	0.08962	1.5560	0.10864	1.1135	0.38636	**4.4025**	**0.00174**	**17.5135**	**0.00042**	**11.7595**	**0.00028**	**25.6205**	**0.00073**	**325.6565**	**0.00001**	**246.8385**	**0.00018**
ABCA10	**0.1424**	**0.01479**	**0.4399**	**0.04091**	0.7866	0.08426	**1.9940**	**0.02313**	**2.7996**	**0.01902**	**7.1054**	**0.01487**	**8.9058**	**0.00155**	**28.4145**	**0.00030**	**115.3137**	**0.00343**	**212.1048**	**0.00011**

Note - Fold change values presented in the table were determined by normalising the gene (mRNA) expression level of each tested MPM cell line to the non-malignant MeT-5A control, as determined via qPCR analysis.
P-values were determined via a Student's t-test. Statistically significant (p-value ≤ 0.05) fold changes in gene expression, with respect to the MeT-5A control, are highlighted in bold.

Table S6. Statistical significance evaluation of upregulated / downregulated gene expression of IAP, drug transporter and survivin-related genes.

Gene (mRNA)	P-Value	Statistically Significant?	Upregulated / Downregulated?
OCT1	0.00819	Yes	Downregulated
MDR1 (ABCB1)	0.18210	No	-
MRP1	0.17800	No	-
BCRP (ABCG2)	0.00829	Yes	Downregulated
IAP1	0.41860	No	-
IAP2	0.02598	Yes	Upregulated
ILF3	0.23060	No	-
BIRC5	4.38600E-06	Yes	Downregulated
ABCG2	0.11770	No	-
XIAP	0.18090	No	-

Note - Statistical significance was determined by comparing the gene (mRNA) expression levels of all tested MPM cell lines in comparison to the non-malignant MeT-5A control via a Welch Two Sample t-test statistical analysis.
Differences in gene (mRNA) levels that produced a p-value of ≤ 0.05 were deemed to be statistically significant.
(-) denotes MPM cell gene (mRNA) levels that were not found to be statistically significantly upregulated or downregulated in comparison to MeT-5A.

Table S7. Statistical significance evaluation of observed increases in MPM cell sensitivity to YM155 treatment of MSTO-211H MPM cells pre-treated with siRNA.

siRNA Treatment	P-Value	Statistically Significant?
si-ABCA6-01	0.1219	No
si-ABCA6-02	4.8509-9	Yes
si-ABCA10-01	0.1403	No
si-ABCA10-02	3.3404E-15	Yes

Note - Statistical significance results presented in the table were determined by measuring the p-value of chi-squared statistical analyses comparing nested sigmoidal models of observed increases in MSTO-211H MPM cell sensitivity to YM155 treatment of siRNA-treated cells with respect to the inactive siRNA control mimic. siRNA treatments yielding a p-value of ≤ 0.05 were deemed statistically significant.
